# Placental Growth Factor for the Prediction of Adverse Outcomes in Patients with Suspected Preeclampsia or Intrauterine Growth Restriction

**DOI:** 10.1371/journal.pone.0050208

**Published:** 2012-11-28

**Authors:** Jeanne Sibiude, Jean Guibourdenche, Marie-Danielle Dionne, Camille Le Ray, Olivia Anselem, Raphaël Serreau, François Goffinet, Vassilis Tsatsaris

**Affiliations:** 1 Cochin Hospital, APHP, Paris-Descartes University, Paris, France; 2 PremUP foundation. Paris, France; 3 DHU Risks and Pregnancy, Paris, France; 4 University of Montreal, Montreal, Canada; 5 INSERM Unit U953, Epidemiological Research Unit on Perinatal Health and Women's and Children's Health, Paris, France; VU University Medical Center, The Netherlands

## Abstract

**Background:**

The circulating concentration of PlGF is reported to be lower in patients experiencing preeclampsia and patients delivering a small for gestational age (SGA) neonate. To evaluate the predictive value of circulating PlGF for preeclampsia and adverse outcome in patients with suspected preeclampsia or intrauterine growth restriction (IUGR).

**Methodology/Principal Findings:**

A double blind prospective study. We enrolled 96 women for suspected preeclampsia or IUGR, and measured plasma levels of PlGF (Triage®) at enrolment. We defined adverse outcome as severe preeclampsia, SGA neonate (<10^th^ centile) or elective delivery for maternal or fetal complication. Severe adverse outcome was studied among patients included <34 weeks gestation (WG) and defined as eclampsia, HELLP syndrome, very SGA (<3^rd^ centile) or elective delivery <34 WG. The mean logtransformed PlGF level was lower for women who experienced preeclampsia (2.9 vs 3.7, p = 0.02), and was markedly lower for patients who experienced adverse outcome (2.9 vs 4.3, p<0.001). The odds of presenting an adverse outcome were higher for the lowest tertile of PlGF compared to the higher (OR = 13 , 95% CI [3–50]). For severe adverse outcome, odds were respectively for the lowest and intermediate tertile as compared with the higher tertile : OR = 216, 95% CI [18–2571]; and OR = 17, 95% CI [3–94]. When included <34 WG, patients with a PlGF level <12 pg/ml experienced a severe adverse outcome in 96% of cases (24/25), and only 1 of 20 patients with a PlGF level >5^th^ centile experienced a severe adverse outcome within 15 days (5%).

**Conclusions/Significance:**

Among women with suspected preeclampsia or IUGR, PlGF helps identify women who will experience an adverse outcome and those who will not within a time period of 15 days.

## Introduction

Preeclampsia (PE) is a multi-system pregnancy-specific disease, affecting 2 to 8% of all deliveries, with a trend towards an increase in recent years [1], [2]. It is a major cause of maternal and fetal morbidity and mortality worldwide, and its management and consequences are responsible for considerable health care expenditure in developed countries [3].

The only curative treatment is delivery. The choice between delivery and expectant management depends on fetal gestational age and maternal conditions. It is important to balance the risks between maternal and perinatal outcomes, as expectant management seeks to improve neonatal outcome, but maternal conditions may worsen [4], [5], [6], [7], [8]. Antenatal care consists of evaluating the severity of the disease and the risk of adverse outcome in order to adapt maternal and fetal monitoring, administer corticosteroids when necessary and refer the patients to a maternity ward equipped with maternal and pediatric intensive care units [9], [10]. By the time preeclampsia has been diagnosed clinically, time to delivery can be very short. Moreover, our capacity to predict severe maternal and perinatal outcomes remains poor [11], [12], [13]Several means of pre-clinical diagnosis and prognostic evaluation have been studied, such as Doppler ultrasound examination of umbilical or uterine arteries and biochemical markers (urinary protein, uric acid), with various degrees of predictive accuracy [14], [15], [16], [17]. This suggests that a pre-clinical diagnostic test able to predict maternal and fetal risk could be useful [18], [19]. Finally, patients who are evaluated for suspicion of preeclampsia undergo many exams, usually during in-hospital care, to assess or rule out the diagnosis of preeclampsia. The associated costs could be reduced if diagnostic discrimination was more accurate.

Among biochemical markers, attention has recently focused on angiogenic factors, such as placental growth factor (PlGF). Key findings support the hypothesis of a pathogenetic model of defective placentation, with consequently reduced concentrations of angiogenic growth factors (free PlGF) and increased concentrations of anti-angiogenic factors (sFLT-1) [20]. Case-control studies have shown a significant drop in maternal free PlGF concentrations in preeclamptic patients compared with nonpreeclamptic patients [21], [22], [23]. Moreover, a study by Levine et al. showed that the drop in free PlGF concentrations preceded the clinical diagnosis by several weeks [24]. A prospective study has recently suggested that the sFLT/PlGF ratio appears better than previous markers for the prediction of adverse outcomes [25]. Several companies have developed tools for PlGF measurement in clinical practice. The Triage © PlGF assay is currently being developed as a bedside test, and immediate results could be helpful in diagnosis and prognosis.

Our objective was to test the predictive accuracy of maternal free PlGF concentration for preeclampsia or pregnancy adverse outcomes in patients with a suspicion of preeclampsia or intrauterine growth restriction (IUGR) based on a single assay at the time of admission.

## Methods

### Study population

We conducted a prospective longitudinal non-interventional double-blind study. From January 2011 to November 2011, we prospectively enrolled all patients at >22 weeks of gestation who were admitted to hospital for suspicion of preeclampsia or IUGR. There was no gestational age limit, but patients had to be enrolled before delivery. The inclusion criteria were: (1) chronic hypertension; (2) gestational hypertension without proteinuria at time of inclusion; (3) isolated proteinuria, ie, without hypertension at time of inclusion; (4) association of low platelets and elevated liver enzymes without hemolysis, hypertension or proteinuria, (5) generalized edema; (6) suspicion of IUGR (estimated fetal weight or abdominal perimeter <10^th^ centile according to Chitty reference curves [26]). Categories (1) to (5) were grouped as “suspected preeclampsia”. Patients either consulted spontaneously or were referred by their physician or another maternity ward. Both singleton and twin pregnancies could be included. Blood samples were collected on the day of hospitalization, or at the latest on the following day, and only once during pregnancy for each patient. Clinicians and medical technologists were blinded to the results of the PlGF assay and the medical charts, respectively. Inclusion in the study did not modify patient's management.

Written informed consent for the study was obtained for each patient. The study protocol was approved by the local ethics committee (CCP-Ile de France 1; ref 12555). The plasma collection was also declared to the French Health Ministry (ID RCB: 2011-A00120-41).

Included patients were followed up in our maternity ward until delivery and during the immediate post-partum period. Diagnoses of preeclampsia, gestational hypertension and chronic hypertension were based on ACOG criteria [27]. Inclusion criteria were defined as follows. Gestational hypertension was defined as a blood pressure > = 140/90 mmHg on two occasions at least 2 hours apart, after 20 weeks of gestation (WG) without significant proteinuria at the time of evaluation, and chronic hypertension was defined as the presence of hypertension prior to 20 WG or known before pregnancy. Isolated proteinuria was defined as > = 300 mg/24 h without hypertension at time of evaluation. Low platelets were defined as <150 G/L, and elevated liver enzymes as aspartate aminotransferase (AST) or alanine aminotransferase (ALT) >2N, eg, >80 IU/mL. Generalized edema was defined as de novo edema of the face, upper and lower limbs.

The hospital policy was to hospitalize for evaluation, generally for 2 days patients presenting with hypertension or other signs in favor of preeclampsia in order to rule out this complication and/or organize the follow up. As for suspicion of IUGR, patients were also generally hospitalized if umbilical artery doppler was abnormal or if fetal heart rate monitoring was non reassuring.

### PlGF assays

Maternal blood was collected in EDTA tubes, centrifuged and tested in the next 3 hours. Plasma was assayed for free PlGF using the Triage PlGF test (Alere, San Diego, CA) and the Triage Meter according to the product insert. It is a fluorescent immunoassay which uses two different murine antibodies against PlGF. Plasma (250 µL) plasma is dropped into the PlGF test cartridge. The cartridge is inserted into the Triage Meter which displays results in 10–15 minutes. Assay range was 12–3000 pg/mL, detection limit was 8 pg/mL, intra-assay coefficient of variation and inter-assay coefficient of variation were 11% and 13%, respectively. Results were expressed in pg/mL and analysts were blinded to the clinical diagnosis.

### Variables

Outcome criteria were defined as follows. Preeclampsia was defined as the association of hypertension and significant proteinuria as defined above, occurring before delivery or in the 7 days post-partum. In patients included for chronic hypertension, superimposed preeclampsia was defined by the occurrence of a significant proteinuria. Severe preeclampsia was defined as preeclampsia plus one of the following: diastolic blood pressure > = 110 mm Hg at any time before delivery; epigastric pain; eclampsia (seizure in a woman without underlying seizure disorder); pulmonary edema; oliguria (<400 mL/24 h); proteinuria >3 g/24 h; uric acid >500 IU/mL; creatinine >100 µg/mL; platelets <100 G/L; lactate dehydrogenase (LDH) >600 IU/mL; HELLP syndrome, or disseminated intravascular coagulation. Mild preeclampsia was defined as preeclampsia without any of the criteria defining severe preeclampsia. Small for gestational age (SGA) was defined as a birthweight <10^th^ centile for gestational age, and very small for gestational age (VSGA) as a birthweight <3^rd^ centile [28].

Adverse outcomes were defined by severe preeclampsia, or SGA neonate or elective delivery for maternal or fetal complication. When mild preeclampsia was associated with neither SGA nor elective delivery, it was not considered harmful and was not included in the definition of adverse outcome [9].

Severe adverse outcomes were defined as one of the following: HELLP syndrome, eclampsia, intrauterine fetal death or termination of pregnancy (for very severe IUGR), VSGA infant, or elective delivery before 34 WG because of maternal or fetal complication.

### Statistical analysis

For quantitative comparisons, PlGF concentrations were log transformed to achieve normality. We used the Student t-test to compare logtransformed PlGF values in patients who did and did not experience preeclampsia, adverse outcome and severe adverse outcome, and used one-way analysis of variance (ANOVA) to compare PlGF values according to the severity of preeclampsia.

For simplicity in clinical use, we used two cutpoints for the study of associations and predictive value. The first cutpoint was the 12 pg/ml low range. The second cutpoint took into consideration the fact that PlGF value changes during pregnancy, and was the 5th centile of the PlGF concentration at each gestational age, as has been described previously : 63 pg/mL (19–23+6 WG); 131 pg/mL (24–28+6 WG); 129 pg/mL (29–31+6 WG); 70 pg/mL (32–34+6 WG); 15 pg/mL (>35 WG). [29], [30] We used single variable logistic regression to compare the risk of developing an adverse outcome or severe adverse outcome in each of the three groups, and multivariable logistic regression, adjusting systematically for maternal age, smoking status, body mass index, and systolic blood pressure at admission.

Logrank tests and Kaplan-Meier curves were used to compare times between presentation and delivery according to these groups.

To determine the value of PlGF for the prediction of adverse outcome and severe adverse outcome, we used receiver operating characteristic (ROC) analysis and calculated areas under the curve (AUCs). We also determined positive and negative predictive values for the different cutpoints cited above, as well as positive and negative likelihood ratios, and 95% confidence intervals. All statistics concerning severe adverse outcome were calculated among patients enrolled before 34 WG (n = 65, 68% of the sample). Twin pregnancies were included in the main analyses.

Finally, we conducted a subanalysis among patients enrolled <32 WG, with a singleton pregnancy and a suspicion of IUGR (<10^th^ centile). We studied the accuracy of PlGF for the prediction of delivery before 34 WG in this subpopulation, by calculating predictive values and likelihood ratios for the 12 pg/mL cutpoint. We compared them to the predictive values and likelihood ratios of umbilical cord doppler resistance index, using the 95^th^ centile as the cutoff value.

All p-values were two-sided and values <0.05 were considered statistically significant. All statistical analyses were performed with the use of STATA 11. [31]

## Results

### Patient characteristics and outcomes (Table 1 and 2)

**Table 1:** Characteristics of patients according to outcome.

Characteristics of patients	Adverse outcome		No adverse outcome		p
Maternal age, y	32.2	±0.8	34.2	±1	0.14
Body Mass Index^a^, kg/m2	23.9	±0.7	26.9	±1.3	0.03
Geographical origin, n (%)					
Mainland France	30	(44.8)	12	(41.4)	0.9
Sub-saharean Africa	17	(25.4)	7	(24.1)	
Other	20	(29.8)	10	(34.5)	
Smoking during pregnancy, n (%)	11	(16.4)	3	(10.3)	0.44
Nulliparous, n (%)	41	(61.2)	14	(48.3)	0.24
Pregnancy, n (%)					
Singleton	57	(85.1)	25	(86.2)	0.88
Twin	10	(14.9)	4	(13.8)	
Gestational age at inclusion, WG	30	±0.55	32.6	±0.96	0.01
Inclusion criteria, n (%)					
Chronic hypertension	12	(17.9)	11	(37.9)	<0.001
Gestational hypertension	11	(16.4)	13	(44.8)	
Isolated proteinuria	3	(4.5)	3	(10.3)	
IUGR	37	(55.2)	1	(3.4)	
Biological abnormality or	4	(6)	1	(3.4)	
Generalized Edema					
Blood pressure at admission, mmHg					
Systolic	131.7	±2.8	130.1	±6.8	0.79
Diastolic	80.4	±1.8	77.4	±4.2	0.43
Uric Acid, IU/mL	295	±15.4	262	±15.8	0.16

a **:** **Body mass index** is defined by weight (kg)/height^2^ (m)

**Table 2:** Pregnancy outcomes.

PREGNANCY OUTCOMES	Mean	Standard deviation
Gestational age at delivery (weeks of gestation)	35.5	±4
Inclusion to delivery time (days)	30	±31
Birthweight (g)	2144	±985
	**n**	**%**
Stillbirths	4	4.2
Termination of pregnancy	2	2.1
Preeclampsia	40	41.7
HELLP syndrome	5	5.2
Mode of delivery (among the 90 live births)		
Vaginal	37	41.1
Cesarean section	53	58.9
Delivery		
<34 weeks of gestation	31	32.3
<37 weeks of gestation	50	52.1
Birth weight (g)		
<1500	29	30.2
<2500	57	59.4
Small for gestational age		
<3rd centile	28	29.2
<10th centile	46	47.9
Hospitalization in neonatology unit (among the 90 live births)	51	56.7
Adverse outcome^a^	67	69.8
Severe adverse outcome^b^ (among the 65 patients included <34 weeks of gestation)	39	60.0

Quantitative variables are expressed as mean ± standard deviation, and categorical variables as number of subjects (n) and percentage (%).

**HELLP**: hemolysis, elevated liver enzymes and low platelets, all HELLP syndrome were full HELLP syndromes (according to the platelet nadir, patients were classified as following : 1 patient class 1, 1 patient class 2 and 3 patients class 3);

a : **adverse outcome** is defined by severe preeclampsia, or birthweight <10^th^ centile for gestational age, or elective cesarean section for maternal or fetal disease;

b : **severe adverse outcome** is defined among patients included <34 weeks of gestation, by HELLP syndrome, or eclampsia, or stillbirth or termination of pregnancy, or birthweight <3^rd^ centile for gestational age, or elective cesarean delivery <34 weeks of gestation for maternal or fetal disease.

We included 96 patients during the study period. Most (N = 58) were included for suspected preeclampsia, and 38 for suspicion of IUGR. Most patients (82/96, 85%) carried a singleton pregnancy (12 were dichorionic twin pregnancies and 2 were monochorionic diamniotic twin pregnancies, for these, delivery was programmed around 38 and 36 WG respectively) and were nulliparous (N = 55, 57%). We compared baseline characteristics according to the outcome. Patients who would experience an adverse outcome had a lower body mass index, were enrolled sooner (30 WG vs 32 WG), and were more frequently included because of suspicion of IUGR (p<0.001, Table 1).

There were 4 intrauterine fetal deaths and 2 terminations of pregnancy for very severe IUGR. Preeclampsia occurred in 40 cases (42%), adverse and severe adverse outcome in, respectively, 67 (70%) and 39 cases (60% of the 65 patients enrolled <34 WG). Preterm delivery occurred for 50 patients (52%). SGA concerned 46 infants (48%), and VSGA 28 infants (29%, Table 2).

PlGF values varied from <12 pg/mL (low range) to 881 pg/ml, with median concentration of 18.1 pg/mL and inter-quartile range (<12 pg/mL–87.7 pg/mL).

### Comparison of PlGF values according to pregnancy outcomes (Table 3)

**Table 3:** PlGF values at inclusion according to pregnancy outcome.

Outcomes	All patients (N = 96)		Suspected preeclampsia* (N = 58)		IUGR (N = 38)	
	log(PlGF)	p (t-test)	log(PlGF)	p (t-test)	log(PlGF)	p (t-test)
Preeclampsia		0.02		0.001		0.11
No	3.7±1.6		4.4±1.5		3.1±1.5	
Yes	2.9±1.4		3.1±1.5		2.2±0.9	
Preeclampsia						
No	3.7±1.6	0.06	4.3±1.5	0.01	3.1±1.5	0.27
Yes, mild	3.1±1.1		3.4±1.1		2.1±0.5	
Yes, severe	2.8±1.6		2.9±1.7		2.3±1.1	
Adverse outcome	N = 96		N = 58		N = 38	
No	4.3±1.4	<0.001	4.3±1.5	0.001	4.1	*
Yes	2.9±1.5		3.0±1.5		2.9±1.4	
Severe adverse outcome†	N = 65		N = 31		N = 34	
No	5.1±1.3	<0.001	5.1±1.3	<0.001	5.0±1.9	0.001
Yes	2.4±1.1		2.0±0.5		2.6±1.1	

PlGF values are expressed as mean ± standard deviation.

* 37/38 patients had an adverse outcome, t-test could not be applied.

† among patients enrolled <34 weeks of gestation.

**adverse outcome** is defined by severe preeclampsia, or birthweight <10^th^ centile for gestational age, or elective cesarean section for maternal or fetal disease; **severe adverse outcome** is defined among patients included <34 weeks of gestation, by HELLP syndrome, or eclampsia, or stillbirth or termination of pregnancy, or birthweight <3^rd^ centile for gestational age, or elective cesarean delivery <34 weeks of gestation for maternal or fetal disease.

Mean PlGF values (after log transformation) were significantly lower in patients who later developed preeclampsia compared with patients who did not develop preeclampsia. Lower PlGF values were also significantly associated with adverse outcome and severe adverse outcome. When all patients were taken into account, PlGF levels could not discriminate severe and mild preeclampsia. We stratified these comparisons according to inclusion criteria (group 1: suspected preeclampsia; group 2: suspected IUGR). In the “suspected preeclampsia” subgroup, there was a significant difference between PlGF values according to the severity of preeclampsia (P = 0.01) (Table 3). Such difference was not found for the “IUGR” subgroup (Table 3).

**Figure 1:**
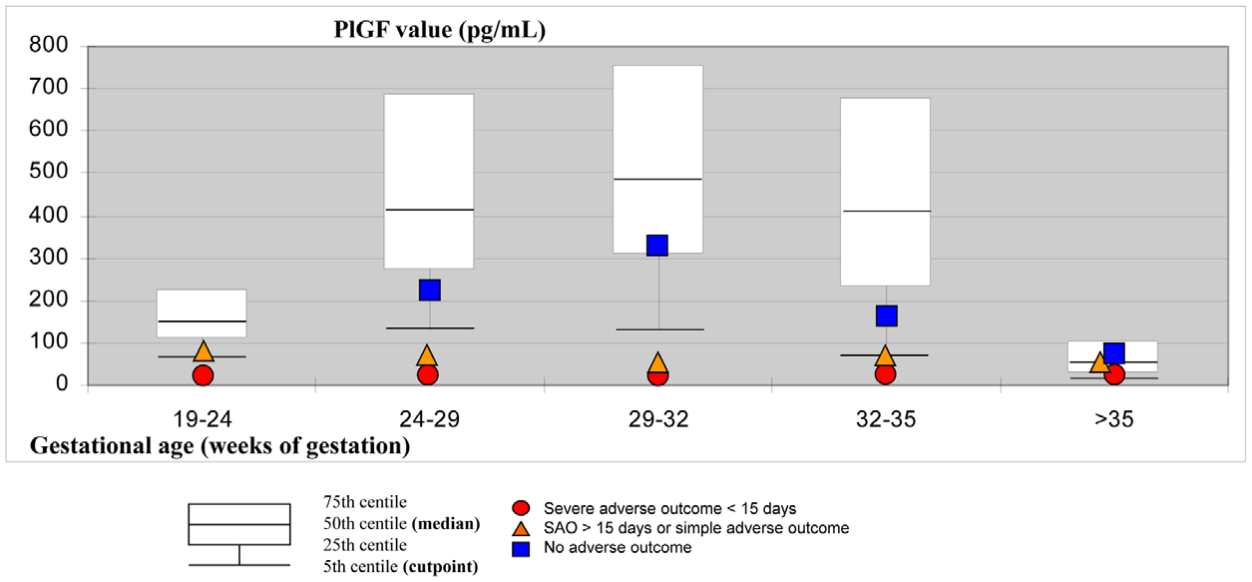
Distribution of PlGF values according to gestational age and pregnancy outcome. SAO: Severe adverse outcome.

Median values of the PlGF at enrolment, according to gestational age and to pregnancy outcome, as well as reference values in a healthy pregnant population are shown in Figure 1, and show that whatever the gestational age, median PlGF value was < = 12 pg/mL for patients who will experience severe outcome in less than 15 days and above the 5^th^ centile for patients who will experience no severe outcome. Median PlGF value was intermediate for patients who will experience severe adverse outcome but in a period >15 days, or a simple adverse outcome.

### Association between PlGF and adverse outcomes (Table 4)

**Table 4:** Association between PlGF and pregnancy adverse outcomes.

Outcomes	%	n/N	OR	95% CI	p	AOR*	95% CI	p
Adverse outcome								
					<0.001			<0.001
<12 pg/mL (low range)	91	31/34	12.9	(3.3–50.1)		15.3	(3.12–74.4)	
12 pg/mL-5^th^ centile	77	20/26	4.2	(1.4–12.9)		7.5	(1.8–31.9)	
>5^th^ centile	44	16/36	1	ref		1	ref	
Severe adverse outcome†								
					<0.001			<0.001
<12 pg/mL (low range)	96	24/25	216	(18–2571)		196	(8–4795)	
12 pg/mL-5^th^ centile	65	13/20	17	(3–94)		26	(2–330)	
>5^th^ centile	10	“2/20”	1	ref		1	ref	

* Multivariable logistic regression, adjusted systematically for maternal age, smoking bmi and systolic blood pressure at admission.

† Among patients presenting <34 weeks of gestation (n = 65).

**adverse outcome** is defined by severe preeclampsia, or birthweight <10^th^ centile for gestational age, or elective cesarean section for maternal or fetal disease; **severe adverse outcome** is defined among patients included <34 weeks of gestation, by HELLP syndrome, or eclampsia, or stillbirth or termination of pregnancy, or birthweight <3^rd^ centile for gestational age, or elective cesarean delivery <34 weeks of gestation for maternal or fetal disease; **OR** : odds ratio; **AOR** : adjusted odds ratio.

When participants were divided into groups according to PlGF value at enrolment, the risk of adverse outcome was greater in the group with PlGF <12 pg/mL compared with the >5^th^ centile group (OR = 12.9, 95% CI 3.3–50.1; p<0.001). Among patients who were included <34 WG, the risk of severe adverse outcome was also higher in the lowest group of PlGF value (OR = 216, 95% CI 18–2571; p<0.001) when compared with the highest group. Results were similar in the multivariable analyses (Table 4).

### Predictive accuracy of PlGF value

**Figure 2:**
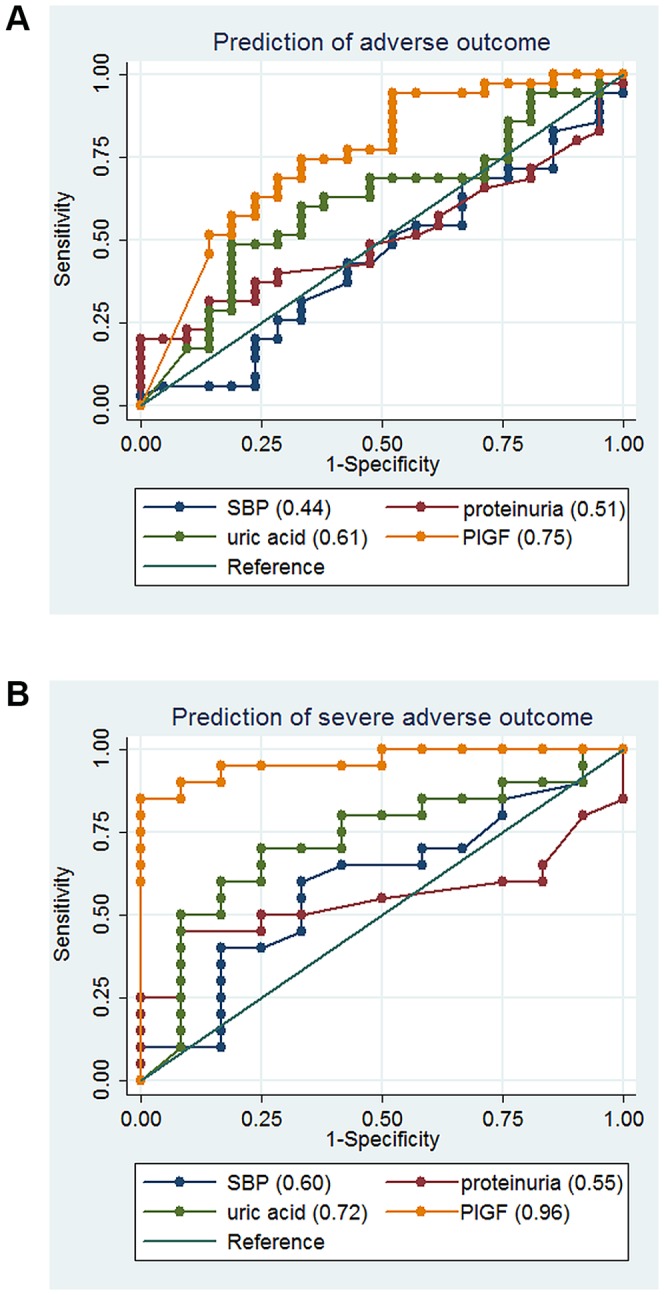
ROC curves for the prediction of adverse outcome and severe adverse outcome. SBP : Systolic blood pressure; PlGF is expressed in pg/mL. Prediction of severe adverse outcome is calculated among 65 patients who were enrolled <34 weeks of gestation. Area under the curve (AUC) values are indicated next to each predictor.

ROC analyses for prediction of adverse outcome and severe adverse outcome were performed and ROC curves are shown in Figure 2. Area under the ROC curves (AUC) was 0.76 for adverse outcome and 0.92 for severe adverse outcome. As a comparison, we performed ROC analyses and calculated AUC for systolic blood pressure, proteinuria and uric acid at admission (Figure 2). None of them performed as well as PlGF; the difference between PlGF and uric acid was significant for severe adverse outcome (p = 0.01), but not for adverse outcome (p = 0.11).

We studied the predictive value of the 12 pg/mL low range cutpoint and of the 5^th^ centile cutpoint. [29] Of 34 patients with a PlGF <12 pg/mL, 31 had an adverse outcome, for a positive predictive value of 91% [81–100], the positive likelihood ratio (LHR+) was 4.47 [95% CI 1.5–13.5]. Among 25 patients included <34 WG with a PlGF <12 pg/mL, 24 had a severe adverse outcome (positive predictive value of 96% [88–100]; LHR+ = 16 [2.3–111]).

Among 60 patients with a PlGF <5^th^ centile for gestational age, 51 had an adverse outcome (positive predictive value: 85% [76–94]; LHR+ = 2.5 [1.4–4.3]), and among the 36 patients with a PlGF >5^th^ centile, 20 had no adverse outcome (negative predictive value: 56% [39–73]; LHR− = 0.3 [0.2–0.6]). As for the prediction of severe adverse outcome, 37 of 45 patients enrolled <34 WG and with a PlGF <5^th^ centile had a severe adverse outcome (positive predictive value = 82% [71–94]; LHR+ = 3.1 [1.7–5.5]) and 18 of 20 patients with a PlGF >5^th^ centile had no severe adverse outcome (negative predictive value = 90% [76–100]; LHR− = 0.07 [0.02–0.3]).

We also studied the occurrence of severe adverse outcome in the 2 weeks following inclusion. In this case, 19 of 20 patients with a PlGF >5^th^ centile had no severe adverse outcome in the 2 weeks following the PlGF sampling: negative predictive value = 95% [83–100]; LHR− = 0.1. The only false negative was in a twin pregnancy who had an elective delivery before 34 WG for anomalies of fetal heart rate in a context of IUGR on one of the twins.

### PlGF value and time to delivery

**Figure 3:**
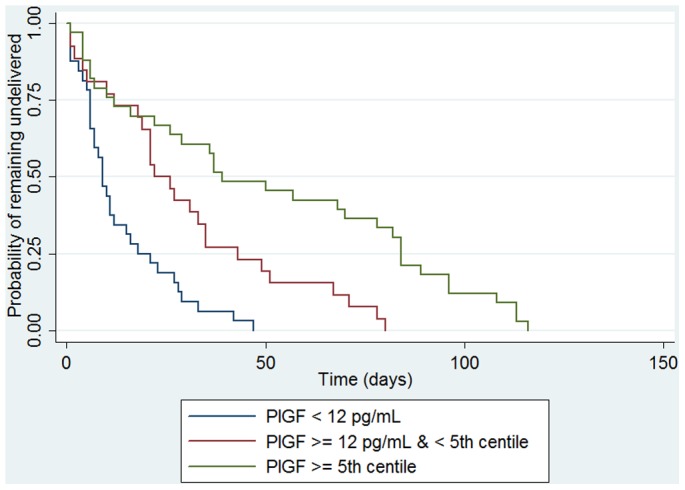
Kaplan-Meier survival curve showing time to delivery according to PlGF. Three groups are compared : PlGF<12 pg/mL (low range), PlGF> = 12 pg/mL and <5^th^ centile, and PlGF>5^th^ centile.

Survival analysis was performed to study relationship between PlGF and time to delivery and showed an association between higher values of PlGF and prolonged time to delivery: the Kaplan-Meier survival curve is shown in Figure 3 (logrank test p<0.001). We found that among singleton pregnancies evaluated before 34 WG and with a PlGF value >5^th^ centile, none were delivered in the next 15 days following the test. In this group, the first delivery occurred 37 days after the test.

We conducted a subanalysis of time to delivery among singleton pregnancies with suspected IUGR, included before 32 WG. Among these 36 patients, 17 had a umbilical cord doppler resistance index >95^th^ centile, 4 had absent flow (RI = 1). In this subpopulation, 17 of 19 patients with a PlGF<12 pg/mL delivered before 34 WG, and 14 of 17 patients with a PlGF>12 pg/mL delivered after 34 WG. The positive and negative predictive values, respectively, 89% [74–100] and 82%[62–100] were higher for PlGF than for umbilical cord doppler, 82% [62–100] and 67% [42–91]. Similarly, positive and negative likelihood ratios were 6.8 [1.8–25] and 0.17 [0.06–0.49] for PlGF versus 3.5 [1.2–10] and 0.38 [0.18–0.78].

## Discussion

Our main result is that we found an association between PlGF concentration used alone and preeclampsia, particularly between decreased PlGF levels and pregnancy adverse outcomes.

Among patients evaluated for suspected preeclampsia or IUGR, a result <12 pg/mL has a high predictive value for adverse outcome (91%). However, the negative predictive value was poor. This may be due to the fact that we had only one blood sample during pregnancy which was sometimes very distant from the complications observed. Although changes in PlGF have been described as preceding the outcome by several weeks [24], it has also been shown that there is a rapid decrease before appearance of preeclampsia, and that the slope is important for the prediction of adverse outcome [32].

When restricting the analysis to patients enrolled <34 WG, PlGF<12 pg/mL had a high predictive value for severe adverse outcome and the 5^th^ centile cutpoint had a high negative predictive value for severe adverse outcome, particularly when it occurred in the next 15 days. We found an association between low PlGF concentration and short time to delivery, as shown by the Kaplan-Meier curve. In the subgroup of patients with suspected IUGR, we studied the probability of delivery <34 WG, which is interesting in order to know which patients to refer to a higher level maternity ward, and also for the indication of administration of corticosteroids. We found high positive and negative predictive values (respectively, 89% and 82%), although large confidence intervals were due to the low number of patients, so this finding must be confirmed in larger studies.

Biochemical markers for preeclampsia have been studied with various objectives.

PlGF or other markers can be helpful to identify if preeclampsia is present if a patient presents with inaugural seizures [33], or in patients with preexisting renal diseases [34], or in patients with thrombocytopenia [35]. Angiogenic factors have also been studied as a screening test early in pregnancy to identify a population at high risk for preeclampsia [36], [37], [38], [39], [40], [41].

Another objective is pre-clinical diagnosis and prognostic contribution which was our purpose in this study. In this non interventional study, PlGF appears to be helpful in anticipating clinical diagnosis, and more importantly predicting the severity of the disease. Only two studies to date have prospectively investigated the value of PlGF in suspected preeclampsia. Rana et al [25] studied the sflt1/PlGF ratio in prediction of a pregnancy adverse outcome. They found results similar to ours, with a positive predictive value of 86% and a negative predictive value of 87% for the cutpoint chosen. The ROC analysis they present shows an AUC of 0.76 , which is also the number we found in our analysis. We found a higher AUC (0.92) when studying severe adverse outcome. The other study investigated the predictive value of various biochemical markers (PlGF, sVEGFR-1 and -2, sEng) in suspected preeclampsia for 87 patients and found a good predictive value of the PlGF/sVEGFR-1 ratio for delivery within 2 weeks [42].

Our study is original because we used only one marker, PlGF, and only once during pregnancy, which is cost-saving in comparison with the two other studies. The Triage © PlGF assay is currently being developed as a bedside test for free PlGF, rendering the aid to diagnosis rapid and comfortable for the patient. Finally, it was a double-blind study and therefore minimizes classification bias.

In conclusion, we found that when patients were evaluated for preeclampsia, the PlGF concentration could predict adverse outcomes accurately. Particularly among pregnant women enrolled <34 WG, the PlGF concentration had high positive and negative predictive values for severe adverse outcome, as well as good positive and negative likelihood ratios, with possible interesting implications for the management of these patients: in-hospital care, transfer to another maternity ward, administration of corticosteroids. Among patients with suspected IUGR, our results suggest an association between low PlGF concentration and short time to delivery, a finding which must be further explored in larger studies. Prospective interventional studies are now needed to assess the real utility of these biomarkers (PlGF alone or sFLT-1/PlGF ratio) in terms of maternal and perinatal outcomes.
